# Interaction Multipath in Through-the-Wall Radar Imaging Based on Compressive Sensing

**DOI:** 10.3390/s18020549

**Published:** 2018-02-11

**Authors:** Yigeng Ma, Hong Hong, Xiaohua Zhu

**Affiliations:** School of Electronic and Optical Engineering, Nanjing University of Science and Technology, Nanjing 210094, China; 311040793@njust.edu.cn (Y.M.); zxh@njust.edu.cn (X.Z.)

**Keywords:** compressive sensing, interaction multipath, ghost suppression, through-the-wall radar imaging

## Abstract

Clutters caused by multipath have been widely researched in through-the-wall radar imaging (TWRI). The existing research work of multipath only consider reflections from the wall, while in the condition of a small scene, with the increasing number of targets, multipath from targets to targets, named interaction multipath, usually generates ghosts, which degrades the performance of TWRI. In order to mitigate the effect of interaction multipath, considering fast data acquisition and measurement reduction, we made use of the propagation characteristic of interaction multipath to build the sparse model of the target scene and developed a compressive sensing (CS)-based method, which is referred to as ‘interaction CS’. For the number of point targets increasing from 5–8, intensive evaluation and direct comparison of the imaging results with existing methods are conducted to show that the proposed interaction CS performs better at ghost suppression in the same condition of the signal-to-noise ratio (SNR).

## 1. Introduction

Due to the potential of revealing targets behind an opaque obstacle, the technology of through-the-wall radar imaging (TWRI) has attracted much interest for public safety and defense applications [1,2,3,4,5,6]. TWRI is especially useful in behind-the-wall reconnaissance, law enforcement and various earthquake and avalanche rescue missions [7,8,9,10]. Existing research of TWRI aims at improving the quality of radar images.

In the theory of TWRI, the interference of clutters is known to be a great challenge for improving the quality of the radar image. As one kind of clutter, the strong reflections of the front wall lead to missed detections of targets behind the wall. Meanwhile, the multiple reflections within the wall result in wall residuals along the range dimension. Another kind of clutter is the multipath caused by multiple reflections off the targets in conjunction with the walls. Multipath delayed returns may generate ghosts, which may be confused with the real targets. Meanwhile, high-resolution imaging demands large aperture and bandwidth; thus a large amount of data needs to be acquired, stored and processed.

Partly in prior work, the challenges mentioned above have been addressed. Clutters caused by the front wall can be addressed by emitting more power, as well as refocusing and wall mitigation techniques [11,12,13,14]. Multipath has been considered as an inverse scattering problem in [15,16]. To address the problem of the large data amount in TWRI, compressive sensing (CS) was first applied by Yoon and Amin [17]. It was shown in [16] and in subsequent publications [17,18,19] that CS is effective in TWRI, if the scene is sparse or can be expressed in a sparse basis. Multipath exploitation with CS was addressed in [20,21], where ghosts caused by interior wall multipath and wall ringing multipath were eliminated. However, the above research work on TWRI only focused on the multipath for reflections from the wall. If there are many strong scatterers with unknown scattering properties in a small scene of interest, interaction multipath, which means the kind of multipath from targets to targets, should also have a greater effect on generating ghosts. In this case, interaction multipath should reduce the quality of TWRI and could not be ignored.

In this paper, we aim at the effect of interaction multipath in TWRI. We made use of the theory of CS for fast data acquisition and measurement reduction and proposed a method for mitigating the effect of interaction multipath. For processing the received signal measurement, we first mitigated the clutters of the front wall with the technique of spatial filtering [11], then modeled the interaction multipath in the over-complete dictionary of the target scene and developed a CS-based method (referred to as ‘interaction CS’) to reconstruct the scene of targets.

In Section 2, the theory of interaction multipath in TWRI is introduced. Subsequently, in Section 3, the method of interaction CS is described. Simulation results are shown in Section 4, and we conclude the paper in Section 5.

## 2. Theory of Interaction Multipath in TWRI

**Figure 1:**
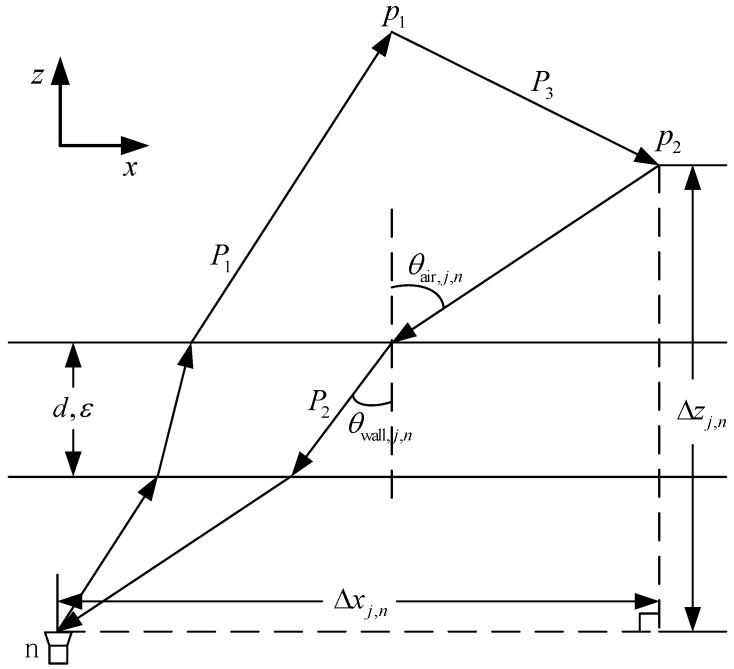
Propagation model of interaction multipath.

We use the stepped-frequency signal that consists of uniformly-spaced frequencies $\left\{f_{m}\right\}$, where m=0,1,…,M−1. A linear array aperture, constituted by *N* wide-band transceivers, is placed parallel to the *x*-axis. Suppose there is a regular grid in the target scene, with the number of grid points, $N_{x}$ and $N_{z}$, representing the cross-range and down-range, respectively. A front wall is at a standoff distance from the array, with thickness *d* and dielectric constant ε. Assume that mono-static operation is adopted and there are two targets, $p_{1}$ and $p_{2}$, in the scene of interest, with the locations of *i* and *j*, respectively, where $i,j\in \{0,1,\ldots ,N_{x}N_{z}-1\}$. The round-trip of interaction multipath is illustrated in Figure 1.

The round-trip consists of path $P_{1}$ from the *n*-th antenna to target $p_{1}$, path $P_{2}$ from target $p_{2}$ to the *n*-th antenna and path $P_{3}$ from $p_{1}$ to $p_{2}$. Therefore, the round-trip delay $\tau_{i,j,n}$ of the signal can be calculated as:

(1) $$\tau_{i,j,n}=\tau_{i,j,n}^{\left(P_{1}\right)}+\tau_{i,j,n}^{\left(P_{2}\right)}+\tau_{i,j,n}^{\left(P_{3}\right)}.$$

It is obvious that if i=j, $\tau_{i,j,n}$ represents the round-trip of one target; if i≠j, $\tau_{i,j,n}=\tau_{j,i,n}$. We assume the thickness and the permittivity of the wall are estimated accurately; therefore, $\tau_{i,j,n}$ can be calculated from geometric considerations.

Thus, the corresponding ideal received signal y[m,n] is the superposition of all the delayed and weighted transmitted signals via i,j, yielding:

(2) $$y\left[m,n\right]=\sum_{i=0}^{N_{x}N_{z}-1}\sum_{j=0}^{N_{x}N_{z}-1}\sigma_{i,j}\exp\left(-j2\pi f_{m}\tau_{i,j,n}\right),$$

where $\sigma_{i,j}$ means the complex reflectivity of the target pair $\left(p_{1},p_{2}\right)$ in the location of i,j. If there exists one target on both *i* and *j*, respectively, $\sigma_{i,j}\neq 0$; else, $\sigma_{i,j}=0$.

For notational convenience, we use the measurement vector $y\in C^{MN\times 1}$ instead of y[m,n], where y is composed by all the measurements y[m,n] and is written as:

(3) $$y={\left[y\left[0,0\right],\ldots ,y\left[M-1,0\right],\ldots ,y\left[M-1,N-1\right]\right]}^{T}.$$

Thus, $\sigma_{i,j}$ can be vectorized as:

(4) $$s={\left[\sigma_{0,0},\ldots ,\sigma_{0,N_{x}N_{z}-1},\sigma_{1,0},\ldots ,\sigma_{N_{x}N_{z}-1,N_{x}N_{z}-1}\right]}^{T}.$$

It is obvious that there is only a small number of nonzero elements in s, which means s is sparse and can be reconstructed with CS.

## 3. Method of Interaction CS

Based on the above-mentioned theory, the model of interaction multipath is feasible to build. First, we need to build the dictionary matrix $\Phi \in C^{MN\times N_{x}^{2}N_{z}^{2}}$, which can be defined as:

(5) $$\Phi_{k+1,N_{x}N_{z}i+j+1}=\exp\left(-j2\pi f_{m}\tau_{i,j,n}\right),$$

where m=kmodM, n=⌊k/M⌋, k=0,1,…,MN−1.

It is certain that the dimension of Φ is too huge, which leads to a large computational complexity. In this paper, we only take (i,j), for which i≥j and r(i,j)≤a, into consideration, where r(i,j) means the distance of (i,j) and *a* is a constant. Because as mentioned in Section 2, $\tau_{i,j,n}=\tau_{j,i,n}$, and if r(i,j) is too large, $\sigma_{i,j}$ is usually too small to be concerned. Thus, we delete the column vectors of Φ: $\left\{\Phi_{:,N_{x}N_{z}i+j+1}|r\left(i,j\right)>a\right\}\cup \left\{\Phi_{:,N_{x}N_{z}i+j+1}|i<j\right\}$ to get the dimension-reduced dictionary matrix $\Phi_{d}$, which significantly reduces the computational complexity. Now, Equation (2) can be written as:

(6) $$y=\Phi_{d}s.$$

It should be clarified that the threshold *a* can be determined according to the current circumstance or be selected empirically. The larger *a* is, the more effectively we mitigate the interaction multipath; the smaller *a* is, the more the computation complexity reduces. With the mentioned method, the amount of the column vectors in $\Phi_{d}$ can be approximately $0.5\pi a^{2}/A_{r}$ of that in Φ, where $A_{r}$ is the area of the target scene.

For stepped-frequency operation, we can use $D\in {\left\{0,1\right\}}^{J\times MN}$, where D is *J* rows of an MN×MN identity matrix. Thus, y can be sampled as:

(7) $$\bar{y}=\mathrm{Dy}=\mathrm{As},$$

where $A=D\Phi_{d}$. We need to reconstruct the reflectivity vector s from measurement Model (7) and use $\left\{\sigma_{i,j}|i=j\right\}$ for imaging the target scene.

It can be observed that the model of our proposed method is similar to the model of the conventional CS method in TWRI. However, the main difference between these two methods is the dictionary matrix. In conventional CS, the dictionary matrix only contains column vectors, each of which has the information of one grid point in the target scene. For interaction CS, based on conventional CS, we have added column vectors, which have the information of considered target pairs, to the dictionary matrix of conventional CS. It is certain that interaction CS with a larger dictionary matrix indeed needs more execution time than conventional CS. In this paper, s is reconstructed by solving the basis pursuit problem [22,23]:

(8) $$\min_{s}{|s∥}_{1}\;\;\mathrm{subject}\;\mathrm{to}\;\;\bar{y}=\mathrm{As}.$$

For noisy measurements, (7) can be rewritten as:

(9) $$\bar{y}=\mathrm{As}+u,$$

where u is the received measurement noise. From [24,25], robust reconstruction of (9) can be achieved by solving the Dantzig selector:

(10) $$\min_{s}{|s∥}_{1}\;\;\mathrm{subject}\;\mathrm{to}\;\;{∥A^{T}\left(\bar{y}-\mathrm{As}\right)∥}_{\infty }<\delta ,$$

where δ is a small tolerance error. In this paper, $l_{1}$-magic [26] is employed for solving (10).

## 4. Simulation Results

Assume we have an $r_{\mathrm{cross}}\times r_{\mathrm{down}}=4\;m\times 3\;m$ room, which has several static point targets with scattering directions uniformly. The scattering coefficients of all targets obey a Gauss random distribution. A uniform linear mono-static array of 77 elements, with an inter-element spacing of 1.9 cm, is posed on the *x*-label; the origin of the coordinate system is at the center of the array. The concrete front wall, with the thickness d=20cm and the relative permittivity ε=7.6632, is located parallel to the array at 1 m downrange. The number of grid points $N_{x}=N_{z}=41$, and the threshold a=0.75 in the simulation. We use a stepped-frequency signal, which consists of 81 equally-spaced frequency steps and covers the 1–3 GHz band. Meanwhile, 1/4 of array elements and 1/8 of frequency bins are used in our experiments, i.e., the compressive ratio is 1/32. The reflections of the front wall, as well as interaction multipath returns are calculated ideally and added to the received signals. It should be clarified that to highlight the interference of the front wall, the reflectivity of the front wall is determined to be 2 in the simulation, and then, the imaging results are normalized.

For the compressed measurement vector ${\bar{y}}_{w}={\left[y_{w}\left[0,0\right],\ldots ,y_{w}\left[M-1,0\right],\ldots ,y_{w}\left[m,n\right],\ldots ,y_{w}\left[M-1,N-1\right]\right]}^{T}$, which includes the front wall clutters, we use the technique of spatial filtering to mitigate the clutters of the front wall and obtain the measurement vector $\bar{y}$. Each element of $\bar{y}$ is expressed as:

(11) $$y\left[m,n\right]=y_{w}\left[m,n\right]-{\bar{y}}_{w}\left(m\right),$$

where:

(12) $${\bar{y}}_{w}\left(m\right)=\frac{1}{N}\sum_{n=0}^{N-1}y_{w}\left[m,n\right].$$

### 4.1. Comparison of Targets’ Reconstruction by Existing Methods and Interaction CS

For comparison, we simulated the method of delay and sum beamforming (DSBF) [20] without processing the clutters of the front wall, and the CS method with the conventional signal model (later referred to as ‘conventional CS’) is also used in the simulation. White noise with a 5-dB signal-to-noise ratio (SNR) is added to the simulated measurements.

**Figure 2:**
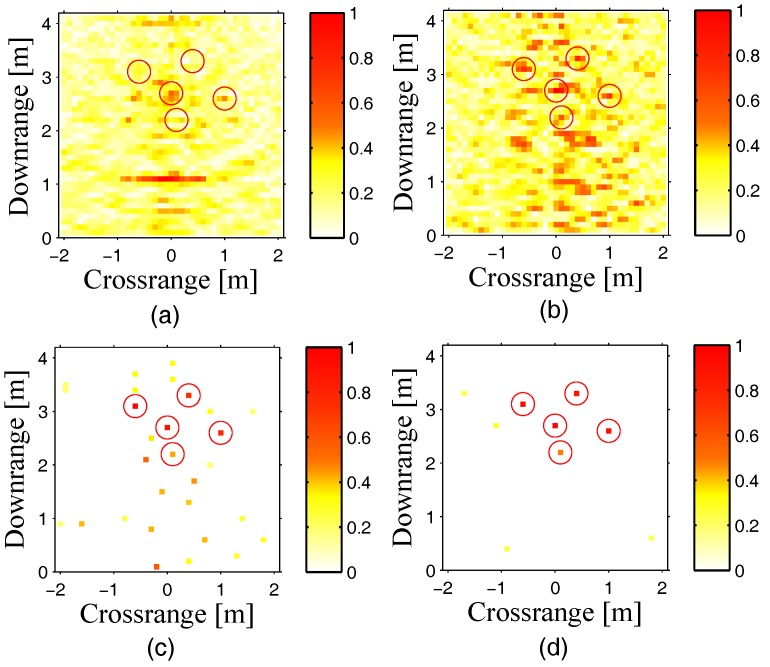
Comparison of targets’ reconstruction by existing methods and interaction compressive sensing (CS). (**a**) For the conventional delay and sum beamforming (DSBF) method without removing any kind of clutters; (**b**) for the DSBF method with front wall clutters removed; (**c**) for conventional CS with front wall clutters removed; and (**d**) for interaction CS with front wall clutters removed.

The normalized imaging results are shown in Figure 2, where the circles represent the locations of real targets. It is observed that if the clutters of the front wall are not removed, as shown in (a), we cannot reconstruct the targets accurately. When the clutters of the front wall are removed, by DSBF and conventional CS, as shown in (b) and (c), all the targets can be reconstructed accurately; however, there still exist many ghost targets in the reconstructed scene. Only by means of the proposed method, as shown in (d), can all the targets be detected accurately, and most ghost targets are removed, which effectively improves the quality of targets’ reconstruction. However, the execution time of (d) is about 30 times the execution time of (c).

### 4.2. Comparison of Targets’ Reconstruction by Increasing the Number of Targets

**Figure 3:**
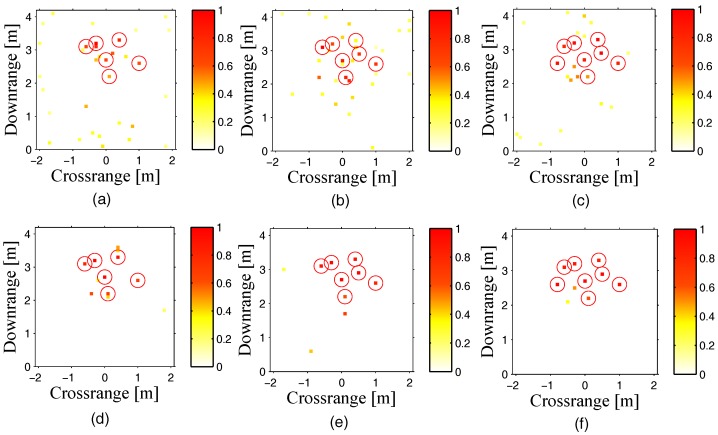
Comparison of targets’ reconstruction by conventional CS and interaction CS with different numbers of targets. (**a**,**d**) for 6 targets; (**b**,**e**) for 7 targets; and (**c**,**f**) for 8 targets; (**a**–**c**) for conventional CS; and (**d**–**f**) for interaction CS. All the front wall clutters are removed.

With increasing the number of point targets from 5–8, we combined the normalized imaging results of both methods with CS, as shown in Figure 3. It can be observed that interaction CS is able to keep a better performance for resisting ghost targets with the increasing number of point targets. For the models of both CS methods, the same as that in Section 4.1, respectively, the difference of the execution time between these two CS methods is similar to that in Section 4.1.

### 4.3. Comparison of Matching Rates in Different Conditions of SNR

**Figure 4:**
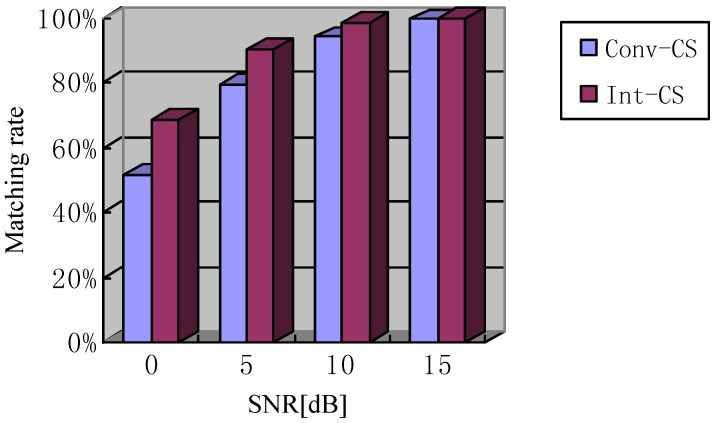
Comparison of matching rates for conventional CS (referred to as ‘Conv-CS’) and interaction CS (referred to as ‘Int-CS’) versus SNR.

Additionally, we combined the matching rates (the ratio of the number of the detected real targets to the number of all real targets) of both methods with CS in different conditions of SNR. The results, averaged with 100 Monte Carlo runs, are listed in Figure 4. The SNR is measured in terms of the average power in the noisy signals, ranging from 0 dB–15 dB. As is seen, the interaction CS seems to perform with a better matching rate.

## 5. Conclusions

In this paper, we deal with the clutter of interaction multipath and have improved the model of the small scene with multiple targets. We modeled this type of multipath in the over-complete dictionary of the target scene and developed the interaction CS method to image the location of targets. For the number of point targets increasing from 5–8, the simulation results showed that the proposed method performs better at removing ghost targets in the same condition of SNR, thereby effectively improving the performance of TWRI. However, the execution of the interaction CS is much slower than that of the conventional CS, and it is not certain whether the slower execution can be compensated by the better image quality in time-sensitive applications. Therefore, it would be useful to simplify the model of interaction CS according to the feature of the target scene, and more efficient reconstruction algorithms and/or their hardware-accelerated implementations can be investigated to speed up the CS methods, including the method of interaction CS.

## References

[1] Ahmad F., Amin M. (2008). Multi-location wideband synthetic aperture imaging for urban sensing applications. J. Frankl. Inst..

[2] Baranoski E. (2008). Through-wall imaging: Historical perspective and future directions. J. Frankl. Inst..

[3] Yoon Y.-S., Amin M.G. (2008). High-resolution through-the-wall radar imaging using beamspace MUSIC. IEEE Trans. Antennas Propag..

[4] Amin M. (2011). Through-the-Wall Radar Imaging.

[5] Tang V.H. (2017). A Sparse Bayesian Learning Approach for Through-Wall Radar Imaging of Stationary Targets. IEEE Trans. Aerosp. Electron. Syst..

[6] Zhang H., Li D., Zhao J. (2017). Time-Delay and Amplitude Modified BP Imaging Algorithm of Multiple Targets for UWB Through-the-Wall Radar Imaging. J. Inf. Process. Syst..

[7] Zhang W., Amin M., Ahmad F. (2012). Ultrawideband Impulse Radar Through-the-Wall Imaging with Compressive Sensing. Int. J. Antennas Propag..

[8] Narayanan R.M., Gebhardt E.T. (2017). Through-Wall Single and Multiple Target Imaging Using MIMO Radar. Electronics.

[9] Li Y.C., Oh D., Kim S. (2018). Dual Channel S-Band Frequency Modulated Continuous Wave Through-Wall Radar Imaging. Sensors.

[10] Tang V.H. (2018). Multipolarization Through-Wall Radar Imaging Using Low-Rank and Jointly-Sparse Representations. IEEE Trans. Image Process..

[11] Yoon Y.S., Amin M.G., Bouzerdoum A. (2009). Spatial Filtering for Wall-Clutter Mitigation in Through-the-Wall Radar Imaging. IEEE Trans. Geosci. Remote Sens..

[12] Tivive F.H.C., Amin M.G., Bouzerdoum A. Wall clutter mitigation based on eigen-analysis in through-the-wall radar imaging. Proceedings of the 17th International Conference on Digital Signal Processing.

[13] Dehmollaian M., Sarabandi K. (2008). Refocusing Through Building Walls Using Synthetic Aperture Radar. IEEE Trans. Geosci. Remote Sens..

[14] TTivive F.H.C., Bouzerdoum A., Amin M.G. An SVD-based approach for mitigating wall reflections in through-the-wall radar imaging. Proceedings of the 2011 IEEE Radar Conference.

[15] Gennarelli G. (2014). Radar Imaging Through a Building Corner. IEEE Trans. Geosci. Remote Sens..

[16] Gennarelli G., Soldovieri F. (2014). Radar Imaging Through Cinderblock Walls: Achievable Performance by a Model-Corrected Linear Inverse Scattering Approach. IEEE Trans. Geosci. Remote Sens..

[17] Yoon Y.S., Amin M. Compressed sensing technique for high-resolution radar Imaging. Proceedings of the SPIE Signal Processing, Sensor Fusion, and Target Recognition XVII.

[18] Ahmad F., Amin M. (2013). Through-the-wall human motion indication using sparsity-driven change detection. IEEE Trans. Geosci. Remote Sens..

[19] Leigsnering M., Debes C., Zoubir A. Compressive sensing in through-the-wall radar imaging. Proceedings of the 2011 IEEE International Conference on Acoustics, Speech and Signal Processing (ICASSP).

[20] Leigsnering M. (2014). Multipath exploitation in through-the-wall radar imaging using sparse reconstruction. IEEE Trans. Aerosp. Electron. Syst..

[21] Gennarelli G., Catapano I., Soldovieri F. (2013). RF/Microwave Imaging of Sparse Targets in Urban Areas. IEEE Antennas Wirel. Propag. Lett..

[22] Candes E., Wakin M. (2008). An introduction to compressive sampling. IEEE Signal Process. Mag..

[23] Chen S., Donoho D., Saunders M. (1998). Atomic decomposition by basis pursuit. SIAM J. Sci. Comput..

[24] Gurbuz A., McClellan J., Scott W. (2009). A compressive sensing data acquisition and imaging method for stepped frequency GPRs. IEEE Trans. Signal Process..

[25] Huang Q., Qu L., Wu B., Fang G. (2010). UWB through-wall imaging based on compressive sensing. IEEE Trans. Geosci. Remote Sens..

[26] Candes E., Romberg J. (2005). L1-Magic : Recovery of Sparse Signals via Convex Programming. http://www.cs.bham.ac.uk/~axk/Sakinah/inspiring_readings/l1magic.pdf.

